# An evaluation of the quality, suitability and impact on equity of clinical practice guidelines relevant to preterm birth for use in Aotearoa New Zealand

**DOI:** 10.1186/s12884-024-06415-0

**Published:** 2024-04-03

**Authors:** Briar Hunter, Lisa Dawes, Makayla Wadsworth, Lynn Sadler, Liza Edmonds, Judith McAra-Couper, Tina Allen-Mokaraka, Katie M. Groom

**Affiliations:** 1 Liggins Institute, University of Auckland, Auckland, Aotearoa New Zealand; 2 Taonga Tuku Iho Principal Investigator Group, Auckland, Aotearoa New Zealand; 3 National Women’s Health, Te Whatu Ora Te Toka Tumai, Auckland, Aotearoa New Zealand; 4 Department of Obstetrics and Gynaecology, University of Auckland, Auckland, Aotearoa New Zealand; 5 Department of Women’s and Children’s Health, Te Whatu Ora Southern, Dunedin, Aotearoa New Zealand; 6 Kõhatu Center for Hauora Māori, University of Otago, Dunedin, Aotearoa New Zealand; 7 Department of Midwifery, Auckland University of Technology, Auckland, Aotearoa New Zealand

**Keywords:** Preterm birth, Clinical practice guidelines, Appraisal, AGREE II, ADAPTE, Implementation, Equity

## Abstract

**Background:**

Preterm birth is a leading cause of perinatal morbidity and mortality and a defining event for pregnant people, infants, and whānau (extended families). Recommendations have been made for a national preterm birth prevention initiative focusing on equity in Aotearoa New Zealand, including the development of a national best practice guide. An understanding of the number and quality of guidelines, and consideration of their suitability and impact on equity is required.

**Methods:**

Guidelines were identified through a systematic literature search, search of professional bodies websites, and invitation to regional health services in Aotearoa New Zealand. Obstetric and midwifery clinical directors were invited to report on guideline use. Identified guidelines were appraised by a 23-member trans-disciplinary Review Panel; quantitatively using the AGREE-II instrument and qualitatively using modified ADAPTE questions. The quality of guidelines available but not in use was compared against those in current use, and by health services by level of maternity and neonatal care. Major themes affecting implementation and impact on equity were identified using Braun and Clarke methodology.

**Results:**

A total of 235 guidelines were included for appraisal. Guidelines available but not in use by regional health services scored higher in quality than guidelines in current use (median domain score Rigour and Development 47.5 versus 18.8, *p* < 0.001, median domain score Overall Assessment 62.5 versus 44.4, *p* < 0.001). Guidelines in use by regional health services with tertiary maternity and neonatal services had higher median AGREE II scores in several domains, than those with secondary level services (median domain score Overall Assessment 50.0 versus 37.5, *p* < 0.001). Groups identified by the Review Panel as experiencing the greatest constraints and limitations to guideline implementation were rural, provincial, low socioeconomic, Māori, and Pacific populations. Identified themes to improve equity included a targeted approach to groups experiencing the least advantage; a culturally considered approach; nationally consistent guidance; and improved funding to support implementation of guideline recommendations.

**Conclusions:**

We have systematically identified and assessed guidelines on preterm birth. High-quality guidelines will inform a national best practice guide for use in Taonga Tuku Iho, a knowledge translation project for equity in preterm birth care and outcomes in Aotearoa.

**Supplementary Information:**

The online version contains supplementary material available at 10.1186/s12884-024-06415-0.

## Background

Preterm birth is a leading cause of perinatal morbidity and mortality and may lead to lifelong disability and poor health [1]. The risk of preterm birth varies both between and within populations, often due to differences in individuals’ risk status, but also by factors such as ethnic and cultural background, place of residence, and socioeconomic status [2–6]. This is clearly seen in Aotearoa New Zealand with a lower rate of preterm birth, as well as higher rates of survival following extreme preterm birth, for European and Asian babies than for their Māori (Aotearoa New Zealand Indigenous/First Nations), Pacific and Indian counterparts [3, 7]. Improving quality and consistency of care with equity as a focus should result in less variation in practice and lead to improved outcomes, especially for those currently experiencing the least advantage.

Clinical practice guidelines have the potential to improve practice and outcomes by summarising and synthesising complexities of evidence, thereby increasing speed and efficiency of clinical decision-making [8]. Furthermore, when applied consistently in practice, clinical practice guidelines should support improved equity in care and outcomes. In order to achieve this, they should contain and/or consider a systematic review of all relevant evidence and be developed in partnership with key stakeholders including the populations most affected by the clinical issue [9, 10]. If equity is to be effectively addressed it must be considered from the outset of a clinical practice guideline’s development, and factored into every step including through implementation and evaluation. Without this consideration, there is a risk that there will be no reduction in differences seen by equity factors, or even worsening of equity issues by only improving care and outcomes for those already experiencing system and other advantage [11].

Improving care and outcomes for preterm birth poses a challenge due to the diverse number and type of conditions contributing to both spontaneous and provider-initiated preterm birth. Clinical practice guidance must consider this broad range of conditions and include prediction and prevention of preterm birth, as well as, preparation and management when preterm birth is inevitable or expected.

In Aotearoa New Zealand, preterm birth and equity of access to care have been identified as priority areas for maternity care practice improvement [3, 12–15]. A stakeholder-led initiative, the Carosika Collaborative, has been funded through the New Zealand Health Research Council to undertake a knowledge translation project for equity in preterm birth care and outcomes in Aotearoa. This project, named Taonga Tuku Iho (Te Reo Māori referring to the legacy of preterm babies) includes the development of a national best practice guide to support consistent and equitable practice. This best practice guide will be developed using the ADAPTE framework; adopting, contextualising and adapting recommendations across all aspects of preterm birth care, using existing high-quality clinical practice guidelines relevant to Aotearoa New Zealand, where available, and formulating new recommendations where no high quality guidelines exist, to provide a single point of reference for national use. This requires an understanding of the quality, suitability and impact on equity of the guidelines that are currently in use or are appropriate for use in Aotearoa New Zealand.

## Methods

This study aims to systematically appraise the quality of clinical practice guidelines relating to preterm birth and to assess their perceived impact on equity and suitability for use in the Aotearoa New Zealand context.

### Guideline selection

Clinical practice guidelines were identified and considered for inclusion from the published literature, relevant professional bodies, and the previously named Aotearoa New Zealand District Health Boards. District health boards were the name given to the regional health services in Aotearoa New Zealand up until July 2022. Before they were disestablished, there were 20 district health boards across the country.

### Systematic literature search

A search strategy was developed and refined (Appendix A). Searches were undertaken of EMBASE, MEDLINE and CINAHL databases (Appendix B). Titles and abstracts were screened by two independent reviewers using the Covidence systematic review reference management tool [16], then full-text articles of references thought to be potentially relevant were retrieved and reviewed. A third reviewer was available if consensus was not able to be reached after initial discussion of any discrepancies.

### Professional bodies search

Relevant professional bodies, colleges and societies were identified by the investigator team alongside an internet search. Professional bodies were included if they were involved with any aspect of preterm birth care or the management of its major risk factors. Included professional bodies were Aotearoa New Zealand specific, Australasian or international with relevance to countries with similar resources to Aotearoa New Zealand. A manual search of the websites of each professional body was undertaken by a single investigator to retrieve potentially eligible guidelines. Two reviewers then independently assessed the full text guidelines for inclusion, with a third reviewer available if consensus was not reached after initial discussion of any discrepancies.

### District health board search

Invitations were sent to the obstetric and midwifery clinical directors at each of Aotearoa New Zealand’s 20 district health boards (assigned regional health services until 2022). Clinical directors were contacted by email and invited to complete a questionnaire about guidelines in use at their institution and requested to contribute these guidelines to the study. If a local guideline was explicitly reported as being based on a national or international guideline then the national/international guideline was also considered to be in use. Written informed consent was obtained from all participants. Non-responders were contacted via email two weeks later and any ongoing non-responders were contacted once by telephone a further two weeks later. Ethical approval was obtained.

### Inclusion criteria

Guidelines could be regional, national, bi-national (Australasian), or international (defined as those developed for use in more than one country and with similar resources and health context to Aotearoa New Zealand). Guidelines were included if they contained recommendations on prediction or prevention of preterm birth, preparation for preterm birth, or the acute management of preterm labour. Guidelines on both spontaneous and provider-initiated preterm birth were included, as well as the conditions and major risk factors that contribute to preterm birth. Guidelines were in English and must have been published, reviewed or updated within the last ten years. If multiple versions of a guideline existed, only the latest version was included. Where further guideline documents were referenced, these were retrieved and assessed alongside their relevant guideline.

### Appraisal

Descriptive data were collected for each guideline by a single investigator. Guideline appraisals were undertaken by 23 members of the transdisciplinary Taonga Tuku Iho Review Panel including midwives, obstetricians, neonatologists, radiologists, consumers, Māori, Pacific and Indian representatives, and individuals with experience in clinical practice guideline development. Review Panel members were invited as representatives of a professional college, society or group including consumer groups and national guideline groups or due to expertise in specific areas such as preterm birth and Māori health. All members provided a disclosure of interest declaration with information on their professional affiliations and previous involvement in guideline development. An on-line meeting provided background information and a briefing on the appraisal process, including provision of the Review Panel Terms of Reference, ADAPTE Toolkit [17], AGREE Trust training exercises [18], AGREE II Instrument User’s Manual [19] and an AGREE II tutorial.

Each guideline was allocated to four reviewers representing a variety of stakeholder groups, with two reviewers (BH and MW) undertaking reviews on every guideline. Guidelines were not allocated to reviewers employed or associated with the organisation responsible for the development of a guideline or involved in its development.

Assessment of quality was made using the AGREE II tool [19] via the online My AGREE Plus appraisal platform [20]. This tool was chosen as it is suggested to be the most commonly applied and comprehensively validated guideline appraisal tool worldwide, and the accepted standard for evaluating clinical practice guidelines [21–24]. The AGREE II checklist includes 23 key items organised into six domains. Each guideline was scored by domain, with an overall assessment score and recommendation for use in clinical practice.

Assessment of applicability and/or acceptability for use in Aotearoa New Zealand was undertaken using a modification of Tool 15 from the ADAPTE Resource Toolkit [17] answered on a secure google form. This tool was chosen as it is specifically designed for evaluating guidelines, as well as being in keeping with use of the ADAPTE framework for the overall development of the national best practice guide. It was modified to allow a broader exploration of perceived impact on equity.

### Outcomes

The primary outcome was the overall number and quality of guidelines relevant to preterm birth in Aotearoa New Zealand; firstly those in current use and and secondly those available but not in use.

#### Secondary outcomes

- The proportion of guidelines in use in Aotearoa New Zealand, that are considered to be high-quality.
- The proportion of guidelines that are available, but not currently in use in Aotearoa New Zealand, that are considered to be high quality.
- The quality of guidelines used by district health boards with secondary compared with tertiary (highest level, subspecialist) maternity and neonatal services. (In Aotearoa New Zealand there are three levels of maternity and neonatal care; primary: >37 weeks, no identified pregnancy complications, secondary: caring for babies born from 32 weeks, tertiary: highest level sub-specialist care, caring for babies born from peri-viable gestations.)
- The perceived impact on equity and suitability of guidelines for the Aotearoa New Zealand context.

Predefined criteria for guidelines to be considered ‘high quality’ were any of:

- Score of > 60% in AGREE II Domain 3 Rigour of Development
- Score of > 60% in AGREE II overall assessment score
- > 50% of appraisers recommend them for use in clinical practice or use with modifications.

### Analysis

Descriptive data were reported for all guidelines. AGREE II scores were compared by district health board use and level of maternity and neonatal services, using the Mann-Whitney U test. The frequency of high-quality guidelines was compared using the Chi-squared test. Descriptive statistics were calculated using Microsoft Excel (version 16.59) and SPSS (version 28.0.1.1) [14]. A p value less than 0.05 was considered significant. Variance between appraisers was calculated by determining the intraclass correlation coefficient for consistency. Thematic analysis of the ADAPTE questions was undertaken using Braun and Clarke methodology [25] by a single investigator, with a group conference involving two other investigators to review and refine themes.

### Ethics statement

Written informed consent was obtained from all participants. The study was approved by the University of Auckland Health Research Ethics Committee on 27/09/2021, reference number AH23265.

## Results

**Fig. 1:**
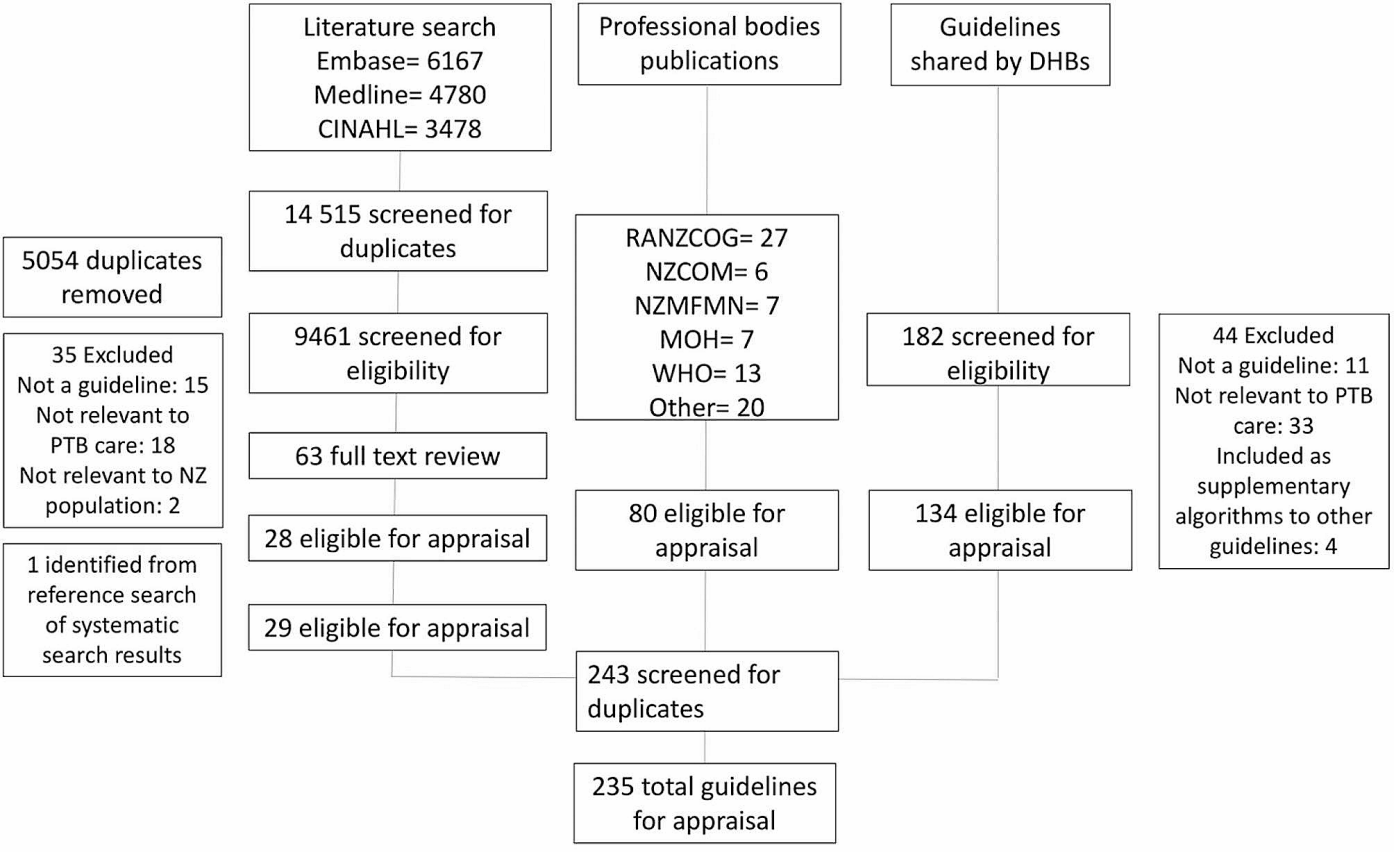
Identified clinical practice guidelines. CINAHL, Cumulative Index to Nursing and Allied Health Literature; RANZCOG, Royal Australian and New Zealand College of Obstetricians and Gynaecologists; NZCOM, New Zealand College of Midwives; NZMFMN, New Zealand Maternal Fetal Medicine Network; MOH, Ministry of Health; WHO, World Health Organization

A total of 235 eligible clinical practice guidelines were evaluated (Fig. 1). Guidelines were shared by 14/20 (70.0%) of Aotearoa New Zealand district health boards, with 13/20 (65.0%) completing the questionnaire on guideline use. Guideline descriptions and their method of identification are reported in Table 1. There were low rates of reporting of funding source (78/260, 30.0%), consumer involvement (63/260, 24.2%), Indigenous involvement in guideline development (11/260, 4.2%) and use of an equity tool (11/260, 4.2%).

**Table 1:** Description of included guidelines

	Literature search *N* = 29	Professional bodies search *N* = 80	District health board search *N* = 151	Total *N* = 260^a^ (%)
**Topic**				
Prediction and prevention of spontaneous preterm birth	6	26	28	60 (23.1)
Prediction and prevention of provider-initiated preterm birth	21	46	73	140 (53.8)
Optimisation and management of preterm birth	2	8	50	60 (23.1)
**Developer**				
Government department	0	7	4	11 (4.2)
Academic institution	0	2	1	3 (1.2)
Colleges, societies and other professional organisations	29	71	12	112 (43.1)
District health boards	0	0	134	134 (51.5)
**Funding source**				
Internal (by developer)	2	31	10	43 (16.5)
External	12	21	2	35 (13.5)
None/not reported	15	28	139	182 (70.0)
**Use of equity tool in guideline development process**				
Equity tool used	3	6	2	11 (4.2)
None/not reported	26	74	149	249 (95.8)
**Consumer involvement in guideline development process**				
Member of guideline development group	1	37	11	49 (18.8)
Consultation	4	9	1	14 (5.4)
None/not reported	24	34	139	197 (75.8)
**Indigenous (Māori) representation in guideline development process**				
Member of guideline development group	0	6	3	9 (3.5)
Consultation	0	1	1	2 (0.8)
None/not reported	29	73	147	249 (95.8)
**Format of publication**				
Guideline	29	49	125	203 (78.0)
Consensus statement/ consensus position statement/ position statement/ best practice statement	0	30	18	48 (18.5)
Manual/protocol	0	1	1	2 (0.8)
Not stated	0	0	7	7 (2.6)
**Region**				
New Zealand	1	25	141	167 (64.2)
Australasian	4	30	7	41 (15.8)
International	24	25	3	52 (20.0)
**Sources of evidence**				
Evidence documented	14	32	9	55 (21.2)
Adapted/adopted/contextualised	0	1	8	9 (3.5)
Not evidence documented/not reported	15	47	134	196 (75.4)
**Currency**				
Within 5 years	16	42	128	186 (71.5)
Older than 5 years	13	38	23	74 (28.5)
**Implementation plan**				
Resources for implementation	12	22	94	128 (49.2)
Implementation plan	7	20	4	31 (11.9)
None	10	38	53	101 (38.8)

^a^ Including 25 duplicates identified across > 1 search strategies

Of the 235 identified guidelines, 151 were reported to be currently in use by district health boards. Another 84 were available but not currently in use in Aotearoa New Zealand. The majority of these were national or international guidelines not identified as being utilised by district health board clinical directors.

The AGREE II quality scores are reported in Table 2. A greater proportion of guidelines that were reported to be available but not in use met the pre-defined criteria for high-quality across every category, than those currently in use (Table 2).

**Table 2:** Quality of guidelines by district health board use

	Guidelines available but not in use by DHBs *N* = 84	Guidelines in current use by DHBs *N* = 151	P value
**AGREE II domains**	**Median scaled domain score** ^**a**^		
Domain 1: Scope and purpose	83.3	70.8	< 0.001
Domain 2: Stakeholder involvement	62.7	37.0	< 0.001
Domain 3: Rigour of development	47.5	18.8	< 0.001
Domain 4: Clarity of presentation	84.0	70.4	< 0.001
Domain 5: Applicability	42.7	29.2	< 0.001
Domain 6: Editorial independence	54.2	4.2	< 0.001
Overall Assessment	62.5	44.4	< 0.001
**Criteria for high quality**	**Number of guidelines meeting criteria for high quality N (%)**		
Median scaled domain 3 score > 60%	30 (35.7%)	7 (4.6%)	< 0.001
Median scaled overall score > 60%	48 (57.1%)	19 (12.6%)	< 0.001
> 50% of appraisers recommended for clinical use	34 (40.5%)	8 (5.3%)	< 0.001
> 50% appraisers recommended for clinical use or use with modifications ^b^	37 (44.0%)	87 (57.6%)	0.036

DHB: District Health Board

^a^Scaled score calculated as: (obtained score - minimum possible score)/(maximum possible score – minimum possible score)

^b^Excludes guidelines already included in the group > 50% of appraisers recommended yes for clinical use

Guidelines reported to be in use by district health boards with tertiary maternity and neonatal services, had higher median scores in the domains Rigour of Development, Clarity of Presentation and Applicability, as well as in Overall Assessment Score, compared with guidelines reported to be in use by district health boards with secondary services (Table 3). There was a greater proportion of guidelines in use by district health boards providing tertiary services that met the high-quality criteria for Overall Assessment Score and recommendation for use in clinical practice or use with modifications compared with district health boards providing secondary services (Table 3). Consistency in AGREE II scores between appraisers was moderate or good for (153/235) 65.1% of guidelines and poor for the remainder.

**Table 3:** Quality of guidelines by type of district health board

	In use by DHBs with secondary maternity and neonatal services *N* = 80	In use by DHBs with tertiary maternity and neonatal services ^a^ *N* = 71	P value
**AGREE II Domain**	**Median scaled AGREE II domain score** ^**b**^		
Domain 1: Scope and purpose	69.4	73.6	0.105
Domain 2: Stakeholder involvement	36.1	38.9	0.442
Domain 3: Rigour of development	14.3	21.9	< 0.001
Domain 4: Clarity of presentation	68.1	72.2	0.004
Domain 5: Applicability	25.0	31.3	0.002
Domain 6: Editorial independence	4.2	4.2	0.619
Overall Assessment	37.5	50.0	< 0.001
**Criteria for high quality**	**Number of guidelines meeting the criteria for high quality N (%)**		
Median scaled domain 3 score > 60%	4 (5.0%)	3 (4.2%)	0.821
Median scaled overall score > 60%	6 (7.5%)	13 (18.3%)	0.046
> 50% of appraisers recommended for clinical use	3 (3.8%)	5 (7.0%)	0.367
> 50% appraisers recommended for clinical use or use with modifications^c^	39 (48.8%)	48 (67.6%)	0.029

DHB: District Health Board

^a^Includes 25 guidelines that are in use by DHBs with secondary and tertiary maternity and neonatal services

^b^Scaled domain score calculated as: (obtained score - minimum possible score)/(maximum possible score – minimum possible score)

^c^Excludes guidelines already included in the group > 50% of appraisers would recommend yes for clinical use

Responses to questions regarding equity and implementation constraints are shown in Table 4. Identified constraints and limitation themes included; cultural considerations in care; service capacity, access to tests, interventions and specialist services; funding to support implementation of recommendations, and staff shortages across the maternity sector (Table 5). Groups identified as experiencing the greatest constraints and limitations to guideline implementation were rural, provincial, low socioeconomic, Māori, and Pacific populations. Major themes of factors that had negative or positive impact on equity were; nationally consistent guidance; culturally considered approach and feasibility of guideline implementation (Table 5). Identified themes to improve equity included a targeted approach to groups experiencing the least advantage; a culturally considered approach; nationally consistent guidance; and improved funding to support implementation of guideline recommendations (Table 5).

**Table 4:** Responses to modified ADAPTE questions

Question	Responses	
	Number (%) (number of guidelines)	
Are there constraints or resource limitations in the New Zealand health care setting that would impede the implementation of the recommendations(s)?	Yes	297 (36.9%) (177 guidelines)
	No	340 (42.3%) (192 guidelines)
	Unsure	167 (20.7%) (127 guidelines)
	Total	804
Do the recommendations in the clinical practice guideline have potential to result in increased or decreased differences in preterm birth outcomes across the population by groupings such as ethnicity, geographic residence and socioeconomic status?	Increased differences	108 (13.7%) (90 guidelines)
	Reduced differences	378 (47.8%) (201 guidelines)
	Unsure of effect on differences	305 (38.6%) (144 guidelines)
	Total	791

**Table 5:** Themes identified from modified ADAPTE questions

Themes	Evidence
**Identified constraints and limitations to implementing the recommendations**	
Limited service capacity	“Constraints on [antenatal clinic] appointments to have women seen in an appropriate timeframe” “Bed capacity to offer admission for all women electing for expectant management [of preterm rupture of membranes]” “Bed availability for inpatient insulin and blood sugar management [for women with diabetes]” “NICU cot availability at local tertiary hospital and need to transfer elsewhere”
Culturally considered care	“Diabetes is over-represented in Māori and Pasifika populations who are also the demographics that suffer disproportionately from inequitable maternity care in NZ”
Access • Geographical constraints • To tests and interventions • To specialist services	“For women in rural areas it may be harder for them to access care when they do start bleeding and therefore do not receive interventions until later “Availability of emergency transfer and retrieval services and neonatal services” “Access to specialist scanning and MFM specialists if required” “BP monitoring more difficult for women living rurally or with transport difficulties. LMCs may have limited ability to visit and perform frequent checks” “Access to ultrasound where demand is exceeding supply and cost [is a] barrier” “Ideally a pre-pregnancy health optimisation consultation would take place for high risk and obese women but the scope and funding for this is certainly not prioritized and more often than not is opportunistic rather than planned. Our highest risk patients are the least likely to present for such consultations secondary to financial social and educational constraints” “Many peripheral centres do not have the neonatal expertise to care for the extreme preterm and women need to be transferred”
Funding	“LMCs not resourced to deliver [anti-D] in the community which may contribute to women not receiving prophylactic dosing” “Funding of LMCs to perform frequent BP monitoring”
Staff shortages	“Maternity services all around New Zealand are facing critical shortages and this means that optimal care is not being provided to women. This is more so in the most unwell women” “National midwifery shortage makes it challenging to perform frequent outpatient BP monitoring as well as undertake staff intensive interventions such as MgSO4 or IV antihypertensive therapy” “There is a shortage of specialist Maternal Fetal Medicine doctors and midwives for the population and this may hinder the advice, support and management that is provided to women”
Education and care coordination	“Consistent availability of treatment recommendations in all main centres” “Staff education around implementation of guideline” “Skill expertise, legislation and policy, and organisational barriers”
**Factors that had a positive impact on equity**	
Quality of guidelines	“Administration of steroids [following binational guideline] to improve outcomes for preterm babies. Universal administration would ideally reduce differences for all” “If recommendations [from binational Magnesium Sulphate guideline] are implemented across all areas, [it] has the potential to reduce differences in PTB outcomes across populations”
Nationally consistent guidance	“The clear guidance for what to do for women who are less than 24 weeks - all women should get [the] same talk and options for their baby” “Good to have clear indications/parameters about when to consider delivery at different gestations [for women with antepartum haemorrhage]” “Good to have explicit information about lines of communication and responsibility plus clear information about funding and support for women and whanau” “Good to have [a] clear step by step guide of how to arrange transfers and who is responsible at each step to streamline this potentially stressful event”
Culturally considered approach	“Good that Māori/Pacific Island ethnicity scores a point as an Amber warning score [on the sepsis guideline] to account for women of these ethnicities often presenting later and having worse outcomes”
**Factors that had a negative impact on equity**	
Quality of guidelines	“Very low quality guideline with no referencing” “Extremely brief guideline, no background, no references, no comment about management”
Nationally consistent guidance	“Recommend more consistency in practice that is likely to vary by clinician and unit”
Culturally considered approach	“There is a lack of cultural safety in how this guideline would apply to Māori and Pasifika patients. As a result, it may be less successful for Māori and Pasifika patients if there is an absence of a more holistic approach, as well as the absence of upstream approaches” “No culturally diverse information/advice provided” “Unless population specific (culturally appropriate) support available then differences between Māori and non-Māori smoking cessation rates are likely to increase”
Feasibility of implementing recommendations	“Women of low SES or with transport difficulties may find it more difficult to access prophylactic dosing and therapeutic dosing of Anti-D due to having to present to hospital to receive this rather than being able to get directly from their LMC in the community” “Access and cost of ultrasound examinations which is more difficult for women who are rural, low SES or have transport difficulties” “Less access to early midwifery care in rural and low SES women. Need to be able to present for consultation and afford prescriptions” “The aim of this statement was to standardise care for peri-viable gestations nationally to reduce equity issues, which is a good step. However the tyranny of distance still applies” “BP monitoring more difficult for women living rurally or with transport difficulties. LMCs may have limited ability to visit and perform frequent checks” “Rural and low socioeconomic populations may face barriers to accessing timely/appropriate clinical care and screening”
**Enablers to improving equity**	
Culturally considered approach	“Prioritisation of culturally appropriate [smoking] cessation support” “Training more Māori and Pasifika midwives” “Whanau-based support rather than individual based given that smoking is often a social activity” “Māori model of quit smoking support” “Programmes to ensure vaccinations can be carried out in [least advantaged] communities by removing barriers to health care. Having Māori and Pasifika role models advocating for vaccinations during pregnancy”
Targeted approach towards groups experiencing least advantage	“Target [preterm labour] guideline to Māori, Pasifika and Indian women who are more likely to suffer greater barriers to antenatal care. This will ensure the implementation of the guideline prioritises the most vulnerable” “Increased funding for sexual health care and treatment for priority (underprivileged/highest incidence) groups” “Consultation and collaboration with target users and population to effect implementation” “Targeted auditing of outcomes in priority groups to provide feedback to clinicians and policy makers as to whether the recommendations are being followed and having effect”
Nationally consistent guidance	“Ensure DHBs use the national guideline with some contextual information only [to apply to local setting]”
Funding to support implementation	“Ensuring reimbursement of travel costs and financial support if women are leaving family behind for many weeks” “Funding of continuous glucose monitoring” “Easier access to Anti-D in the community with protocol and funding for midwives to deliver this where women are” “Loan home BP monitors may help women be able to monitor this more independently”

BP: blood pressure, DHB: district health board, LMC: lead maternity carer, IV: intravenous, MFM: Maternal Fetal Medicine, MgSO4: magnesium sulphate

## Discussion

### Discussion of important findings

**Guidelines that were available but not currently in use by district health boards scored higher in quality than guidelines reported to be in current use**. This is a potential major concern but may be justified in some instances as guidelines that were available but not in use did not always cover the same topics as the guidelines in use by district health boards. Furthermore, some guidelines with higher quality scores were from international organisations, such as the World Health Organization, and may have been judged by clinicians as less applicable to the local Aotearoa New Zealand clinical environment. However, similar results were found in an appraisal of clinical practice guidelines on reduced fetal movements in the United Kingdom; with consistently lower AGREE II scores in local maternity unit guidelines compared with established national guidelines [26]. Overall this makes a compelling argument for a high-quality national best practice guide that is prepared for country-specific context allowing easy adoption and implementation by all local health services.

**Guidelines reported to be in use by district health boards with tertiary maternity and neonatal services had significantly higher median AGREE II scores in several domains, than those with secondary level services.** Again, this is a potential major concern and may contribute to differences seen in care and outcomes by equity factors, in particular by region of residence [27]. Similar findings have been reported in an appraisal of Australasian antenatal corticosteroid guidelines, where institutions providing secondary neonatal services had lower scoring guidelines, than institutions providing tertiary services [28]. Our Review Panel identified that rural and provincial areas have greater constraints and limitations to implementing guideline recommendations. Again this strongly supports the development of a high-quality national best practice guide that is suitable for use for all.

Despite 61.5% of clinical practice guidelines being developed in Aotearoa New Zealand, and 80.0% being developed in either Aotearoa New Zealand or Australasia, Indigenous involvement in guideline development was extremely low (reported in 4.2%). This low rate of inclusion of Māori as tangata whenua (people of the land), combined with a low rate of reporting equity tool use during guideline development (4.2%), is in contrast with the established roles of district health boards and Aotearoa New Zealand professional bodies to uphold the principles of Te Tiriti o Waitangi, a founding document of Aotearoa New Zealand [29]. Having a culturally considered approach to guideline development and implementation was identified by our Review Panel as a key factor to support the ability for guideline recommendations to reduce differences by equity factors. This key factor has been noted in previous studies incorporating equity in guideline implementation [30]. Inclusion of members of the most impacted populations and use of an equity tool from the outset of guideline development have been identified as essential for guideline success in addressing equity issues [30], and have been studied specifically in the Aotearoa New Zealand context [31]. It is possible that broader Māori representation occurred than was reported and that some institutions may detail guideline development processes in supplementary documents that were not provided to our investigator team, however full disclosure of the guideline development processes and stakeholder involvement is a long-established recommendation for clinical practice guideline reporting [9, 19].

Further themes identified in the analysis of qualitative comments on enablers to improve equity were nationally consistent guidance, and improved funding to support implementation of guideline recommendations. The latter theme has been identified elsewhere as a major equity consideration, likely to be relevant worldwide [32].

Although this study was carried out in the Aotearoa New Zealand context, findings are likely to be relevant and of interest to other countries with similar health resources. This is particularly important given the apparent exponential growth in clinical guidelines in recent years and increasing awareness of consideration of equity in all areas of clinical care including the development and use of guidelines.

### Strengths and limitations

This study included a systematic search covering prediction, prevention, preparation and management of preterm birth. The use of a multi-pronged search strategy allowed for identification of a broad range of guidelines, not limited to those formally published in peer-reviewed journals. Inclusion of regional clinical practice guidelines and a questionnaire on guideline utilisation by maternity care providers, has not been captured in previous appraisals of New Zealand’s maternity sector guidelines [33]. District health board engagement was good, increasing the generalisability of the findings.

The AGREE II instrument was chosen as a systematic, validated, and globally-recognised clinical practice guideline appraisal tool, which is appropriate for use across a wide range of guideline subject matters [21–24]. The use of the online My AGREE Plus platform made for convenient coordination of appraisals and straightforward collaboration of a large panel of reviewers. While the platform and built-in user’s manual made the appraisals easy to complete, the platform was limited in its ability to report results; generating individual data files for each guideline. For a project of this size, this resulted in a large amount of manual processing of results, introducing the potential for human error in transcribing data.

A limitation of AGREE II is the lack of established threshold criteria for quality, which is left to individual research groups to determine based on their research question. Domain 3 ‘Rigour of Development’ was chosen as a priority area for this study, as we were interested in identifying guidelines that had methodological strength and a firm evidence basis that could be trusted for use in developing a national best practice guide. ‘Overall Assessment Score’ was chosen as another quality criteria to provide a global assessment score of the guideline. Guideline recommendation for clinical use provided a pragmatic indicator of value in clinical practice, for guidelines that may have practical value that is not necessarily reflected in their AGREE II domain ratings.

A further major limitation of the AGREE II tool is the lack of comment or consideration of equity in guideline implementation, for which it has received criticism previously [10, 34]. For this reason, we included additional questions modified from the ADAPTE Handbook, specifically to look at guideline applicability, acceptability, and impact on equity [17]. This enabled identification of the major themes of constraints and limitations to guideline implementation and identification of guideline factors that impact on equity. Including a qualitative appraisal component allowed the wealth and breadth of experience of our Review Panel to be appreciated and added a depth to the assessment that would not have been possible from quantitative appraisal alone. These insights were invaluable in identifying enablers and barriers for developing a national best practice guide on this topic.

All appraisers underwent training on the AGREE II tool, however despite this, there was significant variation seen between the scores given by different appraisers, with 35% of guidelines having low consistency in the scores given by different appraisers. This is in part a reflection of the subjective nature of AGREE II scoring, with no definitive guidance provided informing the score that should be given when different criteria are met. Variation likely also arose due to the wide range of backgrounds of the Review Panel and the large number of appraisers, although this broad representation is also a strength of the appraisal.

## Conclusions

This was a comprehensive review of clinical practice guidelines on preterm birth available for use and in use in the Aotearoa New Zealand public health system. The mixed methods appraisal had strengths in identifying guidelines of high methodological rigour, as well as potential constraints and limitations to guideline implementation. Major factors contributing to impact on equity were identified; providing invaluable insight for developing a national best practice guide on this topic to be used across Aotearoa New Zealand.

Development of a national best practice guide for equity in preterm birth care is now underway, in partnership with the groups most impacted in Aotearoa New Zealand; with a culturally considered approach and nationally consistent, feasible recommendations. Future research is planned to assess the impact of this best practice guide, through national reporting of preterm birth core outcomes informed by perinatal healthcare professionals and consumers.

### Electronic supplementary material

Below is the link to the electronic supplementary material.

Supplementary Material 1

## Data Availability

The datasets generated or analysed during the current study are not publicly available due to the specifications of the ethics approval for included district health board clinical practice guidelines, but de-identified data are available from the corresponding author on reasonable request.

## Abbreviations

AGREE: Appraisal of Guidelines Research and Evaluation
