# Supplementary material for: An evaluation of the quality, suitability and impact on equity of clinical practice guidelines relevant to preterm birth for use in Aotearoa New Zealand
Source: BMC Pregnancy Childbirth. 2024 Apr 3;24:234. doi: 10.1186/s12884-024-06415-0 (PMC10993582; doi:10.1186/s12884-024-06415-0)
Supplement: Supplementary file 1 — Supplementary Material 1 [file 12884_2024_6415_MOESM1_ESM.docx]

Appendices

Appendix A

Search Strategy for Literature Search of Published Guidelines

((“Premature Birth or Obstetric labour”[Mesh] OR “premature”[mp] OR “labour” [mp] OR “parturition” [mp] OR “gestational age” OR “pre?term birth”[mp] OR “pre?term delivery”[mp] OR “preterm labo?r”[mp]) AND (“Pregnan*” OR “prenantal”[mp] OR “gestation*”[mp] OR “pregnant woman”[Mesh] OR “matern*”[mp] OR “obstetric”[mp] OR “antenatal*”[mp] OR “birth*”[mp] OR “antepartum”[mp] OR “sexually transmitted disease*”[mp] OR “sexually transmitted infection”[mp] OR “Chalmydia”[mp] OR gonorrh?ea”[mp] OR “bacterial vaginosis”[mp] OR “trichomonas”[mp] OR “syphilis”[mp] OR “f?etal infection”[mp] OR “cytomegalovirus”[mp] OR “toxoplasma*”[mp] OR “parvovirus”[mp] OR “diethylstilb?estrol”[mp] OR cervical treatment”[mp] OR “uterine anomal*”[mp] OR “mullerian anomal*”[mp])) AND (“guideline*”[pt] OR “practice guideline”[pt] OR “recommendation”[ti] OR “standard”[ti] OR “guideline*”[ti])

Appendix B

Embase Search


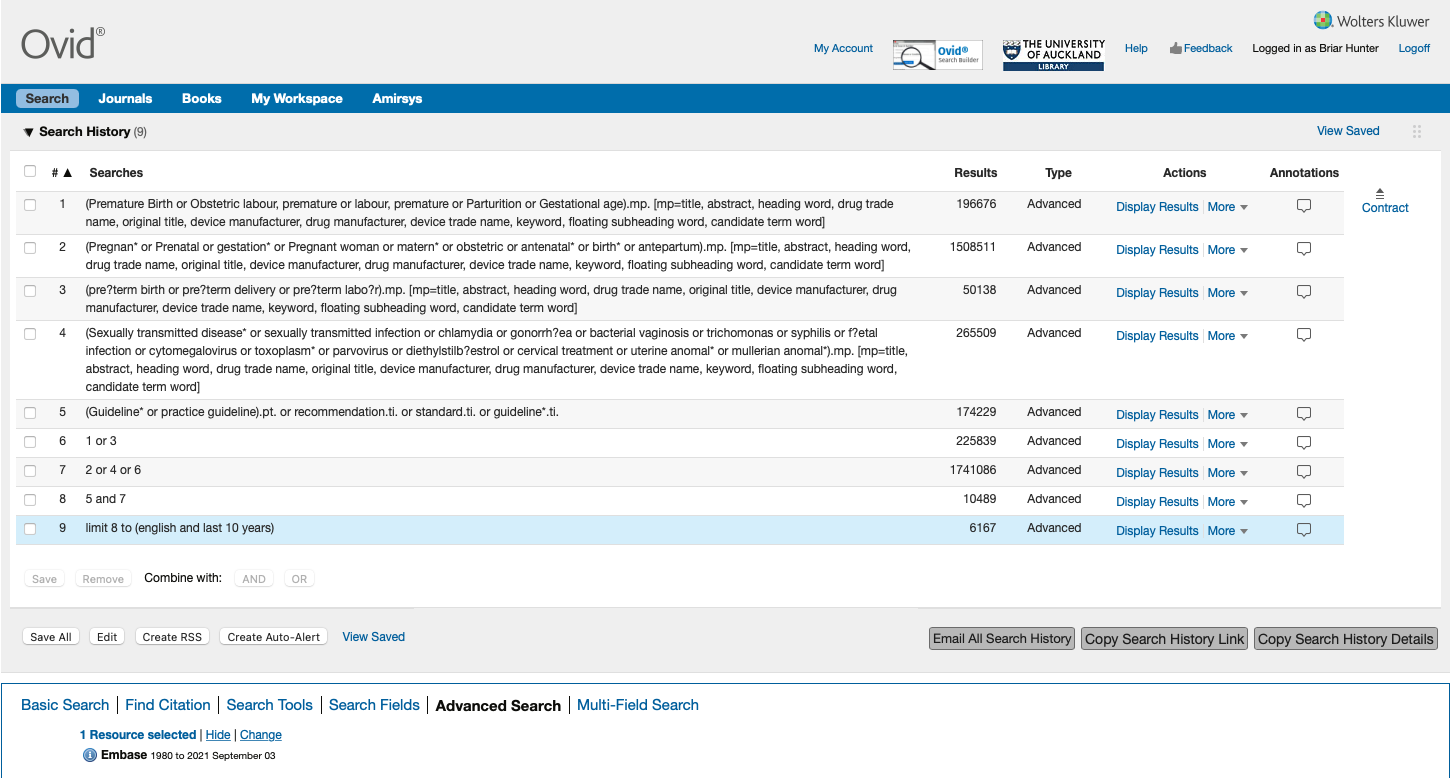


Appendix C

ADAPTE Questionnaire

**ADAPTE Appraisal Questions**

Please complete these questions for each guideline that you appraise.

In this section we really want to hear your expert opinion as specialists and individuals with lived experience of the effects of these recommendations.

If you are representing a professional body on the panel, please feel free to canvas opinion from others within your professional body to better enable you to answer the questions.

These questions will enable us to identify the guidelines that are appropriate for inclusion in a national best practice guidance document, and assess whether they promote achievement of equity in the New Zealand context.

**Your name**

**Name of clinical practice guideline**

1. Was this clinical practice guideline developed specifically for the New Zealand population?

Yes  No

If No, was it developed for an Australasian population?

Yes  No

If No, was it developed for an international population with similar resources to New Zealand?

Yes  No

Are there constraints or resource limitations in the New Zealand health care setting that would impede the implementation of the recommendations(s)?

Consider the following when answering:

Organisational barriers

Legislation and policy

Knowledge expertise

Skill expertise

Interventions

Equipment

Yes  No  Unsure

If Yes, what constraints or limitations exist?

If Yes, is this only applicable in certain areas of New Zealand, and which areas?

1. Do the recommendations in the clinical practice guideline have potential to result in increased or decreased differences in preterm birth outcomes across the population by groupings such as ethnicity, geographic residence and socioeconomic status?

Increased differences

Reduced differences

I am unsure what effect the recommendations would have on different populations

If so, which recommendation(s) within the clinical practice guideline and why?

If the difference may become greater, how may this be overcome?
